# Integrated analysis of human-animal-vector surveillance: West Nile virus infections in Austria, 2015–2016

**DOI:** 10.1038/s41426-018-0021-5

**Published:** 2018-03-14

**Authors:** Jolanta Kolodziejek, Christof Jungbauer, Stephan W. Aberle, Franz Allerberger, Zoltán Bagó, Jeremy V. Camp, Katharina Dimmel, Phebe de Heus, Michael Kolodziejek, Peter Schiefer, Bernhard Seidel, Karin Stiasny, Norbert Nowotny

**Affiliations:** 1 Viral Zoonoses, Emerging and Vector-Borne Infections Group, Institute of Virology, University of Veterinary Medicine Vienna, Veterinärplatz 1, 1210 Vienna, Austria; 2 Blood Service for Vienna, Lower Austria and Burgenland, Austrian Red Cross, Wiedner Hauptstraße 32, 1040 Vienna, Austria; 3 Center for Virology, Medical University of Vienna, Kinderspitalgasse 15, 1090 Vienna, Austria; 4 Institute of Medical Microbiology and Hygiene, Austrian Agency for Health and Food Safety (AGES), Währingerstraße 25a, 1096 Vienna, Austria; 5 Institute for Veterinary Disease Control Mödling, Austrian Agency for Health and Food Safety (AGES), Robert Koch-Gasse 17, 2340 Mödling, Austria; 6 Section Equine Internal Medicine, Equine University Clinic, University of Veterinary Medicine Vienna, Veterinärplatz 1, 1210 Vienna, Austria; 7 Technical Office of Ecology and Landscape Assessment, Nibelungenstraße 51, 3680 Persenbeug, Austria; 8 Department of Basic Medical Sciences, College of Medicine, Mohammed Bin Rashid University of Medicine and Health Sciences, Dubai Healthcare City, Building 14, P.O. Box 505055, Dubai, United Arab Emirates

## Abstract

The results of integrated human and veterinary surveillance for West Nile virus (WNV) infections in Austria during the transmission seasons 2015 and 2016 are shown. Altogether WNV nucleic acid was detected in 21 humans, horses, wild birds and mosquito pools. In detail: in four human clinical cases [two cases of West Nile fever (WNF) and two cases of West Nile neuroinvasive disease (WNND)]; eight blood donors [among 145,541 tested donations], of which three remained asymptomatic and five subsequently developed mild WNF; two horses with WNND, of which one recovered and one had to be euthanized; two wild birds [one goshawk and one falcon, both succumbed to WNND]; and five *Culex pipiens* mosquito pools. Compared to previous years the number of infections increased remarkably. All infections were recorded in the city of Vienna and neighboring regions of Lower Austria. Sixteen coding-complete WNV sequences were established which were closely related to each other and to other Austrian, Czech and Italian viruses, all belonging to the Central/Southern European cluster of WNV sublineage 2d. However, several genetically slightly different WNV strains seem to co-circulate in the same area, as demonstrated by phylogenetic analysis. Based on detailed sequence analysis, all newly discovered Austrian WNV strains had the potential to cause neurological disease, but no correlation was found between severity of disease and the analyzed genetic virulence/neuroinvasiveness markers. Results of integrated human-animal-vector surveillance presented in this paper provide a comprehensive description of WNV activity in the region and will facilitate proactive public health measures to prevent or mitigate potential outbreaks.

## Introduction

West Nile virus (WNV) is a member of the genus *Flavivirus* within the family *Flaviviridae* with almost worldwide distribution. In nature it circulates between wild bird hosts and certain mosquito vectors, mainly *Culex* sp. Humans and horses are considered dead-end hosts. About 80% of infected humans remain asymptomatic while 20% develop mild febrile illness (West Nile fever, WNF); less than 1% of cases present with encephalitis, meningitis or encephalomyelitis (subsumed as West Nile neuroinvasive disease, WNND) of which approx. 10% die^1^. In equids, WNV infection is frequently also asymptomatic or associated with fever, however it may also result in encephalomyelitis with ataxia, paresis and paralysis; in these cases mortality rate may reach 25%^2^. Avian mortality is considered a good early indicator for WNV activity in certain regions. Bird species such as goshawks and other raptors are highly vulnerable to WNV lineage 2 infection in central Europe and commonly develop neuroinvasive disease, frequently with fatal outcome^3–5^. In some areas and/or by infection with other WNV strains avian mortality may be less pronounced or even absent^6^. Of the nine currently suggested WNV genetic lineages^7^ only lineage 1 and 2 are of significant medical importance. Both lineages are considered endemic in central and southeastern Europe. Apart from an outbreak in a horse population in France in 1962^8^, no larger outbreaks caused by WNV lineage 1 were reported in Europe until two subsequent epidemics occurred in Bucharest, Romania in 1996^9^ and in Volgograd, Russia in 1999^10^. WNV lineage 2 was limited to sub-Saharan Africa until 2004, when it was first identified in wild birds in Hungary^11^ and subsequently spread to central^3,4^ and southeastern European countries^5,12^. Meanwhile, WNV is widespread in Europe causing notable outbreaks and sporadic cases of WNF/WNND^13^. Italy, which has been one of the most affected countries in Europe, initiated nucleic acid testing (NAT) of blood supplies in 2008^14^. In 2011 the European Centre for Disease Prevention and Control (ECDC) started to publish weekly updates of affected areas and numbers of confirmed human and – since 2017 – equid WNV cases in Europe^13^. Subsequently, in order to ensure safety and quality of the blood transfusion chain at the European level, a guidance was published at the beginning of 2012^15^.

In Austria, WNV lineage 2 was first detected in wild birds and *Cx. pipiens* mosquitoes in 2008^3,4^. Three human clinical cases were reported retrospectively, dating back to 2009 and 2010, respectively^16^. In 2014, the Austrian Red Cross (ÖRK), Blood Service for Vienna, Lower Austria and Burgenland implemented a seasonal WNV NAT screening of all blood donations, which resulted in the first detection of WNV in a blood donation from Vienna in the same year^17,18^. While fatal West Nile disease (WND) bird cases were reported every season since 2008, up to this publication no WNV infections were documented in the Austrian horse population^19^.

Here we present the comprehensive results of an integrated human-animal-vector surveillance for WNV in Austria during the two transmission seasons 2015 and 2016 including a detailed genetic analysis of the newly discovered viruses.

## Material and methods

### Organization of the integrated human-animal-vector surveillances in Austria

In Austria, human, veterinary and entomological surveillances of WNV include: seasonal surveillance of blood donations originating from endemic areas; detailed sample examination of humans and equids with corresponding central neurological symptoms (CNS); active and passive ornithological monitoring; as well as regular, nationwide mosquito surveillance. Endemic areas are those where WNV activity was previously described or is currently proved. Until now, three federal states of Austria are considered endemic: Vienna, Lower Austria and Burgenland.

All samples originate from routine surveillance activities of the corresponding institute. For example, the human population is monitored through both screening of donor blood collected from WNV-endemic areas by the ÖRK, as well as testing of human clinical cases by the Center for Virology at the Medical University of Vienna (MedUni Vienna). Similarly, samples from sick horses at the Equine Clinic of the University of Veterinary Medicine Vienna (VetmedUni) are tested for WNV under routine diagnostic services by the Institute of Virology at VetmedUni, and verified by the Austrian Agency for Health and Food Safety (AGES). Testing of sick or moribund captive birds submitted by their owners and passive monitoring of dead wild birds is performed by the Institute of Virology at VetmedUni. Finally, AGES supports nationwide mosquito monitoring, with additional sampling in areas of WNV activity as determined by human and animal sampling efforts.

Through close collaboration by several institutions, putative positive human or animal WNV cases are confirmed and reported to national and international authorities, e.g., the Austrian Federal Office for Safety in Health Care (BASG), the Austrian Federal Ministry of Health and Women’s Affairs (BMGF), and the ECDC. Through coordinated efforts, the samples are ultimately transferred to the Viral Zoonoses, Emerging and Vector-Borne Diseases Group at VetmedUni for subsequent analysis. Thus, multiple independently operating agencies act to diagnose, confirm, and report WNV activity in Austria.

### Sample collection

#### Human samples

All blood donations collected by the ÖRK during the WNV transmission seasons from 1st June to 30th November 2015 and 2016 were sent to the German Red Cross, Blood Service for Baden–Württemberg–Hessen, Frankfurt, Germany, for WNV NAT. Blood donations were screened in minipools of 19 samples using the cobas^®^ WNV assay on a cobas^®^ 8800 system (Roche, Rotkreuz, Switzerland).

Positive plasma samples were re-tested at the Center for Virology of the MedUni Vienna, the Austrian National Reference Laboratory for Arbovirus Infections. A total of eight confirmed WNV NAT-positive blood donations (5 from 2015 and 3 from 2016) were submitted to the Institute of Virology, VetmedUni Vienna, for further investigations.

Moreover, four clinical WND cases (2 from 2015 and 2 from 2016) were diagnosed by the Center for Virology of the MedUni Vienna, however only samples of the two patients from 2015 (one plasma sample and one urine sample, respectively) were available for in-depth studies. Thus, altogether 10 human samples could be subjected to detailed molecular and phylogenetic analyses. In addition, urine samples of the second patient were investigated during three months following infection.

WNV serology of all 12 human infections was performed at the Center for Virology, MedUni Vienna. WNV-specific IgM and IgG antibodies were determined with commercially available kits (West Nile *Detect*™ IgM Capture ELISA Kit, InBios, Washington, USA, and Anti-West-Nil-Virus ELISA IgG, Euroimmun, Luebeck, Germany) following the manufacturers’ instructions. The WNV neutralization test (NT) was performed as described previously^16^.

All WNV NAT-positive blood donors, as well as clinical WND cases were notified and relevant personal, epidemiological and infection-related data, including their residences and activities during the last two weeks before blood donation or onset of disease, were recorded.

#### Animal samples

##### Birds

Samples of two birds with immunohistochemical and/or pathohistological suspicion of flavivirus infection were investigated within the framework of routine diagnostic services.

A female goshawk, approx. 4 months old and kept in captivity in Lower Austria, about 20 km north of Vienna, was found dead in August 2015. Pathohistologically mainly nonsuppurative myocarditis and liver cell necrosis were diagnosed. WNV antigen was demonstrated by immunohistochemistry in several organs including brain, spleen, kidney, liver and lung.

An 8-year old female falcon, which was kept in captivity in Lower Austria, approx. 40 km north-west of Vienna, showed apathy, anorexia, disorientation and other neurological symptoms, and died mid-September 2015. Pathohistologically fibrinoid necrosis in the spleen and degeneration of myocard fibers were observed.

Brain and other inner organ samples of both birds were available for virological investigations.

##### Horses

Samples of two horses, which were hospitalized at the Equine University Clinic of the VetmedUni Vienna, were submitted for virological investigations.

A 6-year old Haflinger gelding, originating from an eastern district of Vienna, was hospitalized due to fever and ataxia in the last week of August 2016. The horse responded well to symptomatic treatment and improved rapidly; it was discharged twelve days after admission. Several blood and urine samples were taken during its stay and tested for WNV nucleic acid.

A 10-year old Haflinger gelding, stabled in Lower Austria approx. 25 km east of Vienna, was referred with the initial suspicion of laminitis. The horse was hospitalized, but deteriorated rapidly despite treatment and had to be euthanized end of October 2016 due to progressive neurological signs. Pathohistologically, nonsuppurative encephalitis was diagnosed and – after excluding other relevant notifiable CNS diseases – a flavivirus infection was suspected. WNV was officially confirmed by the Institute for Veterinary Disease Control Mödling (National Reference Laboratory for equine encephalomyelitis, AGES) in January 2017. Brain samples were available for subsequent investigations.

#### Ethics statement

In Austria, WNV infections in humans and equids are notifiable, waiving the need for explicit patient or animal owner consent. Data elements collected for these surveillance activities represented the minimal necessary interference.

All human cases were reported to the BASG and BMGF, which extended the reports to the respective European authorities.

The equine cases were also reported to the BMGF, and submitted to the Animal Disease Notification System of the European Commission. Avian WNV cases are not notifiable in Austria.

#### Mosquito samples

Mosquitoes were collected within the framework of the ongoing Austrian mosquito surveillance program, a collaborative program of the VetmedUni Vienna with the AGES, during the entire study period. Mosquito sampling was performed nationwide, however with emphasis on areas close to homes of WNV-positive humans and on invasive mosquito species^20^. Sampling and morphologic typing of mosquitoes were performed as described previously^18^.

In addition, mosquitoes were also collected at the farm of the first horse with verified WNV infection described above. Before PCR screening, all mosquitoes were pooled to a maximum number of 25 individuals according to species, developmental stage, collection site and date.

### Geographic distribution of WNV-positive samples

In order to show the locations of the WNV-positive cases, a map derived from Open Government Data (OGD) Austria (license CC-BY 3.0 AT)^21^ was edited using the Open Source Geographic Information System (QGIS), licensed under the GNU General Public License (2009), Version 2.18.3^22^.

### Nucleic acid extraction, PCR, sequencing, and sequence analysis

Sample preparation was conducted as described previously^18^. From each sample, 140 µl was processed by automated nucleic acid extraction (QIAcube, Qiagen, Redwood, USA) employing a QIAamp Viral RNA Mini QIAcube Kit (Qiagen) according to the manufacturer´s instructions. All nucleic acid extracts were screened by a published WNV (lin.1 + 2) RT-qPCR^23^. Positive samples were subsequently investigated by various conventional RT-PCRs targeting the complete WNV genomes by employing published primer pairs^11,18^ and, if necessary, newly designed primers specific for each individual sample. Primer sequences (approx. 100 primer pairs) are available upon request. The RT-PCR assays, sequencing reactions and sequence alignments were performed as described previously^18^.

### Determination of pathogenicity and neuroinvasiveness markers

Pathogenicity and neuroinvasiveness markers such as predicted N-glycosylation sites or the occurrence of certain amino acids assumed to be associated with increased virulence were determined as described^18,24,25^.

### Genetic distances

The p-distance and MLH algorithms including bootstrap test (1000 replicates) of the MEGA7 program^26^ were conducted to calculate the numbers of nucleotide and amino acid differences between 16 Austrian complete WNV polyprotein sequences established during this study and 2 sequences from 2014^18^.

### Phylogenetic analysis

Processing of the nucleotide sequences coding for the complete polyproteins was performed as described^18^. Besides the 16 sequences determined in this study, an additional 38 WNV lineage 2 sequences from GenBank were chosen, including newly published representative strains from Austria^18,27^, Serbia (unpublished), Bulgaria^28^, Hungary^29^, Italy^30,31^, and Greece^31^. For better resolution, viruses of the WNV clusters 2a, 2b, and 2c (see^18^) were excluded. The phylogenetic tree was created with the MEGA7 program^26^.

### GenBank accession numbers

The newly described coding-complete Austrian WNV sequences are available from GenBank under accession numbers MF984337-52 (sample names are listed in Table 1).

**Table 1:** Synopsis of autochthonous human WNV infections in Austria during 2015 and 2016

Case number/year	Abbreviation	Blood donor screening	Patient (clinical case)	WNF	WNND	WNV NAT in plasma or serum	Date of first WNV NAT-pos. sample	Days between blood donation or onset of symptoms and follow-up samplings	WNV full genome sequencing/GenBank no.	WNV IgM	WNV IgG	WNV NT
1/2015	**BD1/15**	Yes		No	No	Pos	28.07.2015	0	Yes/MF984337	Neg	Pos	<20
						Neg		5		Pos	Pos	≤20
2/2015	**BD2/15**	Yes		No	No	Pos	04.08.2015	0	Yes/MF984338	Neg	Pos	<20
						Neg		8		Pos	Pos	60
3/2015	**BD3/15**	Yes		Mild	No	Pos	11.08.2015	0	Yes/MF984339	Neg	Pos	<20
						Pos		6		Neg	Pos	<20
						n.t.		21		Pos	Pos	80
4/2015	**BD4/15**	Yes		Mild	No	Pos	12.08.2015	0	Yes/MF984340	Neg	Neg	<20
						Pos		5		Neg	Neg	20
						Neg		21		Pos	Pos	120
5/2015	**BD5/15**	Yes		Mild	No	Pos	24.08.2015	0	Yes/MF984341	Neg	Pos	<20
						Neg		9		Pos	Pos	160
6/2015	**Pa1/15**		Yes	Mild	No	Pos	19.08.2015	3	Yes/MF984342	Neg	Pos	40
						Neg		26		Pos	Pos	480
7/2015	**Pa2/15**		Yes		Yes	Pos	24.08.2015	2	Yes/MF984343	Pos	Pos	40
						Pos		5		Pos	Pos	40
						Pos		12		Pos	Pos	80
1/2016	**BD1/16**	Yes		No	No	Pos	03.08.2016	0	Yes/MF984346	Neg	Pos	20
2/2016	**Pa1/16**		Yes	Yes	No	Pos	17.07.2016	5	No sample	n.t.	n.t.	n.t.
						Pos		28		Pos	Pos	480
3/2016	**BD2/16**	Yes		Mild	No	Pos	23.08.2016	0	Yes/MF984347	Neg	Pos	20
						Neg		11		Pos	Pos	240
4/2016	**BD3/16**	Yes		Mild	No	Pos	13.09.2016	0	Yes/MF984348	Neg	Pos	<20
5/2016	**Pa2/16**		Yes		Yes	Neg	-	10	Not applicable	Pos	n.t.	160
						Neg		12		Pos	n.t.	160

*n.t*. not tested

## Results

### General results

**Fig. 1:**
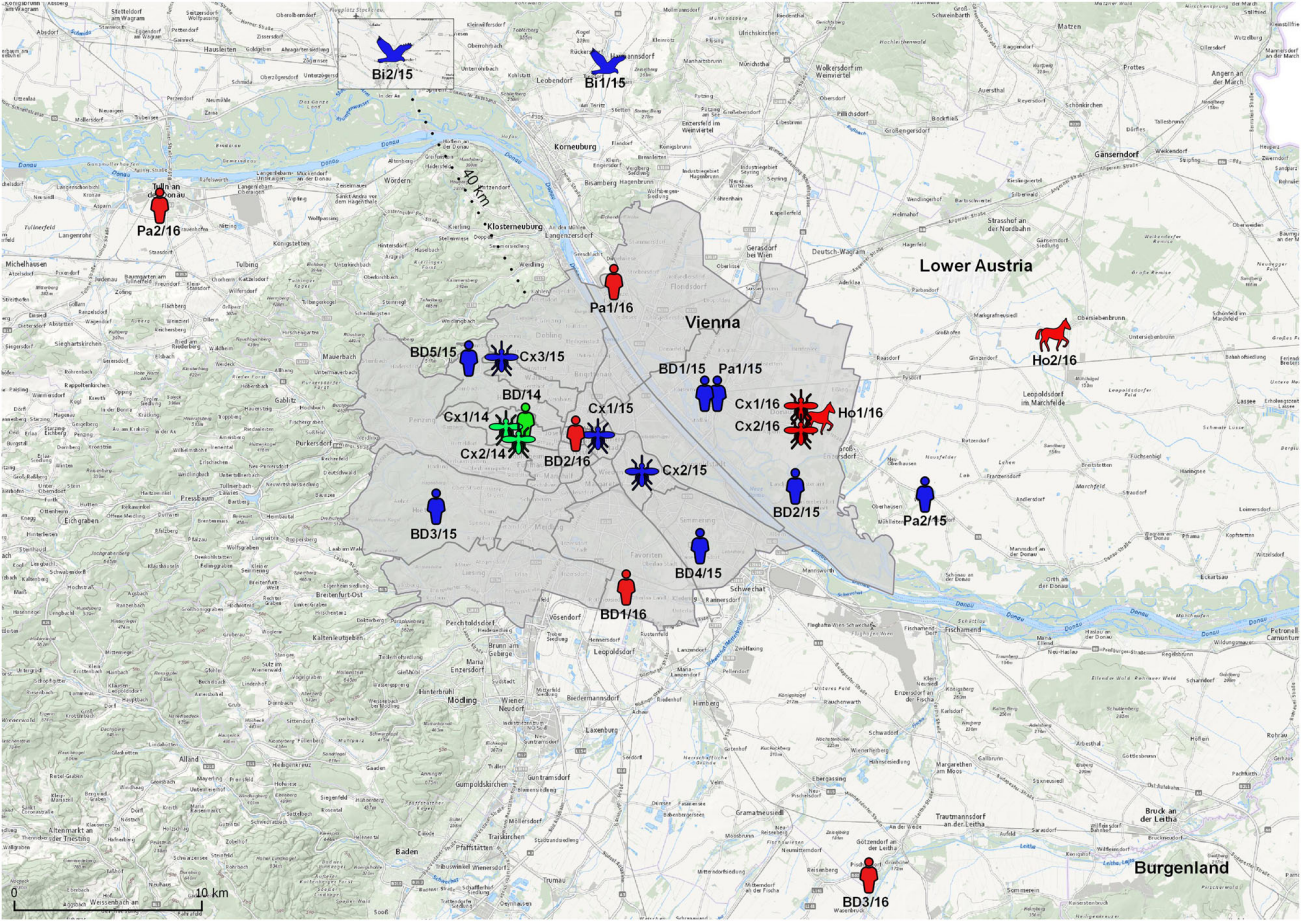
Map showing the geographic distribution of WNVs identified in Austria during the transmission seasons 2014–2016. Green symbols indicate WNV cases in 2014^18^; blue symbols in 2015 (this paper) and red symbols in 2016 (this paper)

Altogether 16 WNV infections in humans and animals, as well as 5 WNV-positive mosquito pools were identified in Austria during the WNV transmission seasons 2015 and 2016 – all of them in the city of Vienna and adjacent areas of Lower Austria (Fig. 1). During 2015 seven human infections (5 WNV-positive blood donors, 1 clinical case of WNND and 1 clinical case of WNF), 2 fatal bird cases and 3 WNV-positive mosquito pools were documented. During 2016 five human cases (3 WNV-positive blood donors, 1 clinical case of WNND and 1 clinical case of WNF), 2 horse cases (1 case of WNF/WNND and 1 fatal WNND case – confirmed retrospectively in January 2017), and 2 WNV-positive mosquito pools were recorded.

The following abbreviations were used throughout the text: BD1/15-BD5/15 and BD1/16-BD3/16 for blood donors, Pa1/15, Pa2/15, Pa1/16 and Pa2/16 for clinical cases, Bi1/15 (goshawk) and Bi2/15 (falcon) for birds, Ho1/16 and Ho2/16 for horses, and Cx1/15-Cx3/15, as well as Cx1/16 and Cx2/16 for *Cx. pipiens* s.l. mosquitoes. Consistently, three positive samples from 2014^18^ were abbreviated: BD/14, Cx1/14, and Cx2/14.

### Human infections

The WNV NAT screening of blood donations revealed WNV nucleic acid in 5 out of 74,677 donations in 2015 and in three out of 70,864 donations in 2016, thus the annual incidences were calculated as one in 14,935 and one in 23,621, respectively.

Of the eight WNV-positive blood donors, three were asymptomatic (BD1/15, BD2/15, and BD1/16) and five developed mild WNF a few days after the blood donation (BDs3-5/15, BD2/16, and BD3/16). BD3/15 reported mild cephalea two days after the blood donation and fatigue for three days; BD4/15 had mild cephalea at day three after the donation and fatigue for two days, and developed an exanthema at day 5; and BD5/15 showed fatigue and an exanthema from day 2 to 5 after the donation. BD2/16 suffered from massive muscle and joint pain and an exanthema one day following the donation, BD3/16 reported muscle pain at the day of the blood donation, but also suffered from sore throat and rhinitis. In all cases, the symptoms were mild and therefore none of the blood donors consulted a physician. WNV infection was confirmed by detection of WNV RNA and sequence analyses. In 6 out of 8 donors follow-up serum samples were available and infection was serologically confirmed by detection of WNV-specific IgM and neutralizing antibodies (Table 1).

Of the four clinical cases, two showed WNF (one in 2015 with mild WNF and one in 2016 hospitalized with WNF) and two developed WNND (also one in 2015 and one in 2016). WNF patient Pa1/15, 62-year female, spouse of BD1/15, developed myalgia and rash three weeks after the blood donation of her asymptomatic husband tested WNV-NAT positive. WNF patient Pa1/16, 27 years, was hospitalized with high fever and fatigue. He was treated with antibiotics and discharged from hospital healthy after 4 days. WNND patient Pa2/15, 82-year male, was hospitalized with fever and neurological signs. He developed encephalitis and tetraplegia and was treated in an intensive care unit. The patient survived with severe sequelae. WNND patient Pa2/16, 75 years, was diagnosed with meningitis and could be discharged without any residual neurological problems.

All blood donors and patients reported mosquito bites shortly before blood donation and/or onset of disease.

WNV infection was confirmed in all clinical cases by detection of WNV-specific IgM and neutralizing antibodies. WNV-specific IgM was additionally detected in the cerebrospinal fluid (CSF) of both cases with WNND. In 3 patients WNV RNA was detected in serum or plasma (Table 1). In the case with WNV encephalitis/paralysis (Pa2/15), WNV RNA was also present in CSF and urine, in the latter for a period of 90 days after onset of symptoms. In contrast, WNV RNA could not be detected anymore in serum and CSF obtained 10 days after onset of symptoms from Pa2/16 with WNV meningitis.

The virus loads of the human serum/plasma and CSF samples were relatively low (Cq values between 33.2 and 39.0). Only in the urine sample of Pa2/15 (with WNND) high amounts of WNV RNA were detected (Cq value 27.3).

### Animal WND cases

#### Birds

Samples of brain and pooled other inner organs of the goshawk (Bi1/15), as well as the brain sample of the falcon (Bi2/15) tested highly positive by the screening RT-qPCR with Cq values of 14.2, 19.0, and 15.4, respectively. Whole genome sequences were generated from 1:100 diluted brain extracts from both birds.

#### Horses

Of the 17 blood and urine samples investigated by the WNV (lin.1 + 2) RT-qPCR, only two blood samples of the recovered horse Ho1/16 taken on the second and third day after admission were found positive with Cq values of 36.5 and 37.5, respectively. While the first sample was not available for further investigations, generation of the complete genomic WNV sequence was achieved from the second sample.

Of the euthanized horse Ho2/16, five of 6 tested samples (brain stem, cerebellum, hippocampus, cerebrum, and basal ganglia) revealed positive results by the screening RT-qPCR (average Cq values between 31.8 and 39.1); only the thalamus sample was negative. For determination of the complete WNV genome sequence the sample with the highest virus load (brain stem) was used.

In this paper we report both coding-complete sequences of the horse-derived WNVs. The results of detailed clinical investigations of these two equine cases – the first WND cases in horses in Austria – will be published separately.

### Mosquitoes

In 2015 a total of 7,713 mosquitoes were collected and merged to 841 pools. The sampled mosquitoes belonged to the following genera: 6584 *Culex* (686 pools, 85.4%), 871 *Aedes* (112 pools, 11.3%), 177 *Culiseta* (23 pools, 2.3%), 65 *Anopheles* (18 pools, 0.8%), and 16 *Ochlerotatus* (2 pools, 0.2%). WNV RNA was detected in three pools, all consisting of *Cx. pipiens* adults or larvae. Thus the minimum infection rate (MIR = proportion of positive pools per 1000 mosquitoes) calculated for *Culex* only was 0.46, and when considering all mosquito species collected in 2015 it was 0.39. The positive samples were: a pool with 3 adults from downtown Vienna, 1st Viennese district captured on 18.12.2015 (Cx1/15), a pool with 10 larvae from the 3rd Viennese district collected on 03.09.2015 (Cx2/15), and a pool with 10 larvae from the 18th Viennese district trapped on 15.10.2015 (Cx3/15). Due to low virus loads (average Cq value > 38), none of the above mosquito samples could be investigated in detail.

In 2016 a total of 4,571 mosquitoes were collected in all Austrian federal states between September 9th and October 8th, and pooled in 455 vials: 3,291 *Culex* (307 pools, 72.0%), 1,167 *Aedes* (122 pools, 25.5%), 64 *Culiseta* (11 pools, 1.4%), 36 *Anopheles* (10 pools, 0.8%), 8 *Ochlerotatus* (4 pools, 0.2%), and 5 undeterminable species (1 pool, 0.1%). None of these mosquito pools were WNV RNA-positive.

In addition, 270 mosquitoes were collected in and around the stable where horse Ho1/16 was most likely infected: 141 mosquitoes (11 pools, 52.2%) were identified as *Anopheles*, 125 as *Culex* (9 pools, 46.3%), and 4 as *Coquillettidia richiardii* (1 pool, 1.5%). Genetic identification (*Ace2* gene) of single mosquito larvae taken from standing water around the stable revealed that both *Cx. pipiens pipiens* and *Cx. torrentium* were present at the site (data not shown). Two pools exhibited high amounts of WNV RNA by screening RT-qPCR (Cq values of 28.3 and 32.1, respectively). Each of the two positive pools consisted of 16 adult *Cx. pipiens* (Cx1/16 and Cx2/16); they were captured by gravid trap on 26.08.2016. The MIR for captured mosquitoes at this site and time point (extended to 1000 mosquitoes) was 7.41 and for all mosquitoes collected in this year 0.41.

### Geographic distribution of the WNV-positive samples

Figure 1 depicts locations/residencies of the WNV-positive cases and mosquitoes in Austria, 2014–2016. While the habitations of the horses and birds and the locations of the trapped mosquitoes could be determined quite accurately, for the majority of humans – due to possible movements – only the most likely exposure sites are indicated. As the map shows, most of the infections were identified in various districts of the city of Vienna, and only few in neighboring Lower Austria.

### Sequence analyses

In total, 16 complete WNV genome sequences could be generated: 8 from blood donations (BD1/15-BD5/15 and BD1/16-BD3/16), 2 from blood or urine samples of human patients (Pa1/15 and Pa2/15), 2 from brain samples of birds (Bi1/15 and Bi2/15), 2 from blood or brain samples of horses (Ho1/16 and Ho2/16) and 2 from *Cx. pipiens* mosquito pools (Cx1/16 and Cx2/16).

WNV’s single open reading frame (ORF) of 10,305 nucleotides translated to a 3434 amino acid sequence of the putative polyprotein precursor was determined for all 16 Austrian viruses. In addition, a 92 nt long stretch at the 5′-untranslated region (5′-UTR) was generated for all investigated virus strains, as well as 261–616 nt long fragments of the 3′-UTR; the latter varied in size depending on sequence quality and available sample quantity.

The exact number of nucleic acid and amino acid differences were calculated for all Austrian WNV coding-complete sequences identified between 2014 and 2016. They are displayed in Table 2. The nucleotide differences varied from 5 (between BD2/15 and Ho1/16) to 57 (BD3/16 vs. Cx1/16). In one case (BD2/15 vs. Ho1/16) the amino acid sequences were 100% identical to each other, while the remaining sequences differed by up to 12 (BD3/15 vs. Cx2/16) amino acids. The nucleotide and amino acid differences between the viruses derived from the couple (BD1/15 and Pa1/15) were 9 and 2, respectively. The number of nucleotide and amino acid differences between WNVs derived from the convalescent horse and the two mosquito pools collected on the same farm were 27 and 2 for Ho1/16 vs. Cx1/16, and 7 and 2 for Ho1/16 vs. Cx2/16, respectively.

**Table 2:** Sequence difference count matrix of the complete polyprotein sequences of 18 Austrian WNV strains determined between 2014 and 2016

**Strain**	**BD/14**	**Cx1/14**	**BD1/15**	**BD2/15**	**BD3/15**	**BD4/15**	**BD5/15**	**Pa1/15**	**Pa2/15**	**Bi1/15**	**Bi2/15**	**BD1/16**	**BD2/16**	**BD3/16**	**Ho1/16**	**Ho2/16**	**Cx1/16**	**Cx2/16**
**BD/14**	–	5	4	6	10	3	5	6	6	5	4	6	3	4	6	5	6	8
**Cx1/14**	33	–	3	5	9	2	6	5	5	4	3	5	6	6	5	4	5	7
**BD1/15**	31	16	–	4	8	1	5	2	4	3	2	4	5	5	4	3	4	6
**BD2/15**	38	37	35	–	10	3	7	6	2	3	4	6	7	7	0	1	2	2
**BD3/15**	41	40	38	41	–	7	11	10	10	9	8	10	11	11	10	9	10	12
**BD4/15**	34	7	19	38	41	–	4	3	3	2	1	3	4	4	3	2	3	5
**BD5/15**	23	36	34	41	44	36	–	7	7	6	5	7	6	6	7	6	7	9
**Pa1/15**	30	17	9	34	37	18	33	–	6	5	4	6	7	7	6	5	6	8
**Pa2/15**	37	36	34	5	40	37	40	33	–	3	4	6	7	7	2	1	2	4
**Bi1/15**	37	36	34	19	40	37	40	33	18	–	3	5	6	6	3	2	3	5
**Bi2/15**	29	16	14	33	36	17	32	13	32	32	–	4	5	5	4	3	4	6
**BD1/16**	40	27	25	42	47	27	43	24	41	41	23	–	7	7	6	5	6	8
**BD2/16**	12	39	37	44	47	40	29	36	43	43	35	46	–	5	7	6	7	9
**BD3/16**	27	42	40	47	50	43	30	39	46	46	38	49	33	–	7	6	7	9
**Ho1/16**	41	42	40	5	46	43	46	39	10	24	38	47	47	50	–	1	2	2
**Ho2/16**	38	37	35	6	40	38	41	34	5	19	33	42	44	47	11	–	1	3
**Cx1/16**	48	47	45	22	51	48	51	44	21	29	43	52	54	57	27	22	–	4
**Cx2/16**	44	45	43	8	49	46	49	42	13	27	41	50	50	53	7	14	30	–

The numbers of nucleotide and amino acid differences between the virus strains are indicated below and above the diagonal, respectively

Most of the nucleotide substitutions in the individual sequences were unique and did not result in amino acid changes (data not shown); however, one distinct amino acid mutation E-T159A was identified in four Austrian strains: BD/14, BD5/15, BD2/16, and BD3/16 (Table 3). In addition, two distinct amino acid mutations in the E (T157A) and NS2A (A112V) proteins, as well as several nucleic acid substitutions within the 3′-UTR were identified for 7 sequences: BD2/15, Pa2/15, Ho1/16, Ho2/16, Cx1/16, Cx2/16, and partially Bi2/15 (Table 3). One unique amino acid substitution in the NS1 protein (Y35H) was found in the sequence of human patient Pa2/15 with WNND (not shown). Of note, the sequences of the couple BD1/15 and Pa1/15 showed a common unique mutation in the NS4B protein (S14G) (Table 3). A unique mutation in the NS5 protein (E638K) was also observed in the blood donor strains BD/14 and BD2/16, whereas BD3/16 had glycine at this position (E638G) (Table 3).

**Table 3:** Amino acid mutations in different proteins and nucleic acid substitutions within the 3′-UTR of 18 Austrian WNV-positive samples

	

Unique substitutions are indicated in red. Non-shaded cells, subcluster 1; rose-shaded cells, subcluster 2; green-shaded cell, single sequence in subcluster 3; grey-shaded cells, subcluster 4. Point mutations occurring in only single sequences are not included

Mutations predicted to influence virulence, pathogenicity or neuroinvasiveness were analyzed as described recently^18,24,25^. All Austrian strains exhibited the potentially more virulent proline at position NS1-250. Moreover, the N-glycosylation motif NYS at positions 154–156 of the E protein and three potential N-glycosylation sites at positions 130, 175, and 207 within the NS1 protein, all molecular determinants of neuroinvasiveness, could be identified in all strains as well. All strains shared the more virulent alanine at position NS2A-30, but the less virulent histidine at position NS3-249.

Furthermore, all Austrian strains had threonine at position NS1-338, serine at positions NS2A-126 and NS3-421, as well as phenylalanine at NS5-254, all being characteristic for the more virulent genotype of WNV lineage 2^24^. Moreover, at position E-159 threonine was found in most Austrian strains except BD/14, BD5/15, BD2/16, and BD3/16, where alanine was identified (Table 3).

### Phylogenetic tree

**Fig. 2:**
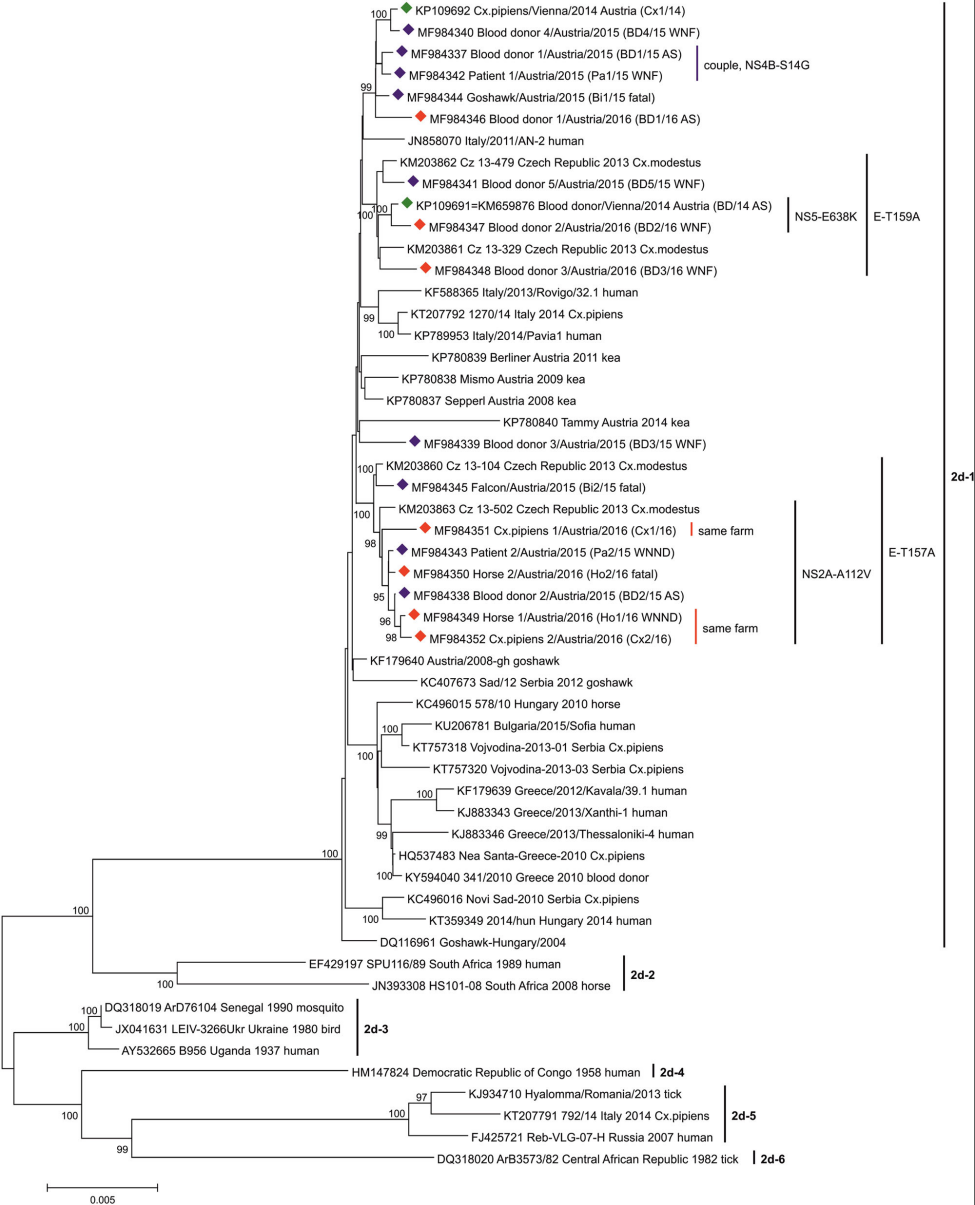
Phylogenetic tree of 54 selected complete polyprotein-coding nucleotide WNV sublineage 2d sequences. Green diamonds indicate the 2 sequences from 2014^18^. Sixteen viruses determined in this study are marked with blue diamonds (identified during 2015) and red diamonds (2016). The six major clusters (1–6) of WNV subclade 2d are indicated by vertical bars. All Austrian viruses belong to the Central/Southern European cluster 2d-1. The GenBank accession numbers, strain names and – if not included in the strain names – geographic locations, years of identifications and host species names are indicated at the branches. For the Austrian strains, common mutations and symptoms (AS/WNF/WNND/fatal) are also depicted. Supporting bootstrap values ≥90% are displayed next to the nodes. The horizontal scale bar indicates genetic distances (here 0.5% nucleotide sequence divergence). Abbreviations used: BD, blood donor; Pa, patient; Ho, horse; Bi, bird; Cx, *Culex* mosquito; AS, asymptomatic; WNF, West Nile fever; WNND, West Nile neuroinvasive disease

Phylogenetic analyses with different statistical methods resulted in very similar cladograms. The highest bootstrap values were achieved by application of the neighbor-joining (NJ) method and the p-distance statistical model. The phylogenetic tree of WNV sublineage 2d exhibits six different clades (Fig. 2). While the recent viruses belonging to clade 2d-1 originate from Central/Southern European countries, the strains of clades 2d-2, 2d-3, 2d-4, and 2d-6 are mostly of African origin. The WNV prototype strain B956 (Uganda 1937) clusters in clade 2d-3. Clade 2d-5 consists of South-Eastern European WNVs.

All newly identified Austrian strains are distributed among 4 subclades of clade 2d-1 (Fig. 2), which evolved from the common ancestor sequence WNV Hungary/2004 goshawk^11^ (GenBank no. DQ116961). The first subclade consists of exclusively Austrian sequences derived from humans (3 blood donors and one clinical case), one bird and one mosquito pool, all collected between 2014 and 2016. The second subclade is made up of 4 Austrian human viruses (all 4 blood donors), also detected between 2014 and 2016, and 2 Czech mosquito sequences discovered in 2013 (GenBank nos. KM203861-2). The distinct blood donor sequence BD3/15 shares the third subclade with the sequence of an Austrian kea which died in 2014 (GenBank no. KP780840). The last subclade consists of two Czech mosquito sequences from 2013 (GenBank nos. KM203860 and KM203863) and 7 Austrian WNV strains detected in different species during 2015 and 2016: two humans (one WNND case and one blood donor), two horses (one euthanized and one survived), one bird (falcon, which died), and mosquitoes (two *Cx. pipiens* pools collected in the farm of the horse which survived). The sequences of the couple (BD1/15 and Pa1/15) are placed close to each other within the first subclade. The sequences of the recovered horse Ho1/16 and one pool of mosquitoes (Cx2/16) collected in its stable are very closely related, while remarkably the sequence of the other mosquito pool trapped essentially at the same place (Cx1/16) is more distant.

## Discussion

Integrated surveillance analysis gave us the unique opportunity to gain more insight into the WNV strains circulating in Austria including clinical features and genetic diversity. In order to achieve this goal, we established 16 whole genome sequences obtained from the main species involved in the transmission cycle and analyzed them extensively.

An Austrian national WNV Task Force including representatives responsible for public and animal health, as well as for vector monitoring from all affected provinces was established in 2013^19^. In 2014, the members recommended NAT screening of all blood donations originated from endemic areas in Austria, for which an appropriate guideline was established^32^. In parallel, a routine investigation was introduced for WNV infections in all human patients with fever and one symptom of neuroinvasive disease (e.g., aseptic meningitis, or encephalitis)^16^. Since 2015, all confirmed WNV infections of humans are mandatorily reportable^19^. Retrospective analyses revealed serological evidence for three human clinical cases of WNV infection in Austria in 2009 and 2010, respectively^16^. Between 2011 and 2013 no human autochthonous WNV infections were reported^19^. The first confirmed human WNV infection in a blood donor in Austria was identified in 2014 immediately after introduction of the NAT screening^17,18^. During the following two years the number of reported human WNV infections increased remarkably (*n* = 12) – as shown in the current study. This increase could be attributed to the introduction of intense screening of blood donations originating from certain areas considered endemic in Austria. It is also likely that increased attention of clinicians to this emerging disease improved the surveillance system sensitivity in the following years. However, it is not excluded that virus activity was simply stronger during the study period.

Dead-bird surveillance is an excellent way of identifying areas of WNV circulation^3–5,11^. In Austria, it has been carried out since the emergence of the virus in 2008^19^. Although WNV has been detected in over 300 species of birds worldwide^33^, goshawks and other birds of prey, as well as owls were found to be most vulnerable for the lineage 2 strain of WNV circulating in Austria^3,4^. The WNV whole genome sequences of two naturally infected birds analyzed in this study – a goshawk and a falcon, both died due to WNND in 2015 – did not exhibit striking genetic differences when compared with other WNV sequences.

For horses, a national serological screening program for WNV was introduced in 2011^19^. In addition, all equine encephalomyelitis cases were tested for WNV. Until 2016, however, no WNV infections were reported in the Austrian horse population^19^. The first two equine WNV infections in Austria occurred in August and September 2016, respectively. While the first horse recovered, the second had to be euthanized due to advanced WNND. Whole genome WNV sequences were generated from both horses. Sequence analysis did not reveal specific genetic differences between the corresponding WNV strains which could explain the different clinical courses. In contrary, the sequences were phylogenetically closely related to each other and to sequences obtained from mosquitoes collected in the farm of the first horse. Both horses were of the same breed and of similar age, and received immediate veterinary attention, thus, probably other factors contributed to the fatal outcome of one of the equids.

In Austria, active country-wide mosquito surveillance has been regularly conducted since 2011 including mosquito species identification and laboratory testing for various pathogens including WNV. While over 65 mosquito species have been implicated in the transmission of WNV, in Europe *Culex* spp. mosquitoes have been identified as the primary vectors^2^. These mosquitoes are predominantly ornithophilic and are considered not only efficient enzootic (bird-mosquito-bird) but also epizootic (bird-mosquito-human) vectors^1^. In our study, the most abundant mosquito genus was *Culex* followed by *Aedes*. WNV RNA was only detected in *Cx. pipiens* s.l. mosquitoes. The average MIR calculated for the Austrian mosquitoes collected during the study was rather low (0.40), however selective mosquito trapping in areas with documented WNV cases resulted in a 10-20-fold increase of this value, as shown in this study for Ho1/16 and the mosquitoes captured in its stable and in the blood donor case of 2014^18^. In the neighboring Czech Republic WNV was also found in *Cx. modestus*, however with much lesser MIR (0.12)^34^. Of note, WNV strains derived from Austrian or Czech mosquitoes are represented in each of the main Austrian sequence groups, confirming their involvement in the transmission cycle in the various regions.

Detailed phylogenetic analyses revealed placement of all sixteen newly discovered WNV strains within subclade d-1 of lineage 2, together with previously determined Austrian strains, which was not surprising, because they were derived exclusively from autochthonous cases. However, due to the extension of the phylogenetic analysis by adding new strains, four separate Austrian sequence groups could be identified, indicating co-circulation of various WNV genotypes in Vienna and surrounding regions. This genetic variability was already noted in 2014, when two WNV strains derived from a blood donor (BD/14) and mosquitoes collected in close proximity to the donor’s home (Cx1/14) proved genetically different^18^. In the phylogeny established for the current study, these sequences cluster in 2 different sequence groups (Fig. 2). Detailed genetic analysis revealed that these two strains differ at the significant position 159 of the E protein. Usually lineage 2 strains have a valine or isoleucine at this position, however alanine was found in BD/14, as well as in three other blood donations sampled in subsequent years (BD5/15, BD2/16, and BD3/16), but not in Cx1/14 (Table 3). Alanine at this position is characteristic for WNV sublineage 1b strains considered to be of low virulence (e.g., Kunjin virus)^24^. Although none of the WNV-infected blood donors developed more than mild WND, the significance of this amino acid substitution for attenuation of virulence/pathogenicity requires further evaluation. This unique mutation was also identified in sequences derived from two Czech mosquito pools (CZ 13-479 and CZ 13-329) from 2013^34^ which are also part of this subcluster (Fig. 2).

In contrast, in Cx1/14 and the remaining Austrian strains threonine was identified at this position (E-159, Table 3) which was found in WNV sublineage 1a strains considered to be highly neuroinvasive (e.g., NY99, HU03, IT11)^24^, as well as in all Italian WNV lineage 2 strains which emerged in 2011 and 2013–2014^31,35^. Nevertheless, also in these cases, no common effect on disease severity could be observed.

All WNV strains from Austria and neighboring countries carried histidine at position NS3-249 instead of the putatively more virulent proline. At almost all other analyzed genomic marker positions, however, the Austrian strains displayed amino acids which are characteristic for virulent strains of sublineage 2d, which includes important European strains such as VLG07 (=RUSV07), GRE10 and ITA11b^24^. Although fatal avian and equine cases occurred, the newly discovered Austrian strains caused either inapparent infection or only mild disease in most of the affected humans; serious WNND was observed only in elderly patients (average age 78.5 years), which is in line with literature^1^.

The WNV strain obtained from blood donor BD3/15 with mild WNF was rather different and occupied a unique position (third subcluster) together with a WNV derived from a kea in human care, which succumbed to chronic WND in 2014 following a 6-year long illness^27^ (Fig. 2). Since the blood donor’s residence is only 3 km distant from the keas’ aviaries, geographic proximity may in this case be an explanation for the observed genetic similarities (Figs. 1 and 2).

Two other amino acid mutations at positions E-157 and NS2A-112, as well as several nucleic acid mutations within the 3′-UTR (Table 3) were unique and most likely the reason for the formation of a fourth Austrian subcluster. This subcluster was made up by WNV strains from humans and horses with different infection courses, as well as from *Cx. pipiens* mosquitoes (Fig. 2), all originating from the eastern and northeastern areas of Vienna and Lower Austria (Fig. 1). The WNV strain derived from the Austrian falcon (Bi2/15), which died at a location approx. 15 km distant to the Austrian-Czech border in 2015 (Fig. 1), as well as WNVs from two Czech mosquito pools (Cz 13-104 and Cz 13-502) from 2013^34^, are also located in this group (Fig. 2), suggesting an ongoing transnational virus circulation and exchange.

Gene position NS5-638 also seems to be remarkable. While most of the lineage 2 strains exhibited glutamic acid at this position, in BD3/16 glycine, and in BD/14, as well as BD2/16 lysine were identified. Glycine and lysine were found in one Greek strain (Greece/2013/Xanthi 2) and one Italian strain (Italy/2013/Rovigo/32.1), respectively^31^. Additionally, a unique amino acid substitution at position 35 of the NS1 protein was determined in only one human case with WNND (Pa2/15). Tyrosine, which appeared in all other Austrian strains, was exchanged by histidine in this strain (i.e., NS1 Y35H). Interestingly, this amino acid substitution was conserved in all 8 North-Italian strains belonging to the Lombardy cluster mainly derived from WNND cases^31^. This emphasizes that shared unique mutations are present in Austrian, Italian and Greek WNV strains, which can be presumably associated with their common ancestry^11,24^. The exact role of the unique mutations in the Austrian strains has not been described; it could represent an interesting subject for further investigation.

Noteworthy, WNVs derived from one of the 3 asymptomatic blood donors (BD1/15) and his spouse (Pa1/15), who developed WNF three weeks after her husband tested WNV NAT-positive, were genetically similar but not identical (Fig. 2). Although possible sexual transmission of WNV was recently reported^36^, infection by the same mosquito population appears to be more likely in this case. Excretion of WNV in urine probably does not pose a risk for virus transmission, although viral RNA was detected in urine samples of WNV NAT-positive blood donors^37^, as well as in patients with WNND^29,37^. The viral load in urine was higher in neuroinvasive than in febrile patients, and WNV RNA in urine of patients with symptomatic infections was detectable for a longer time after onset of symptoms and in higher quantity than in serum^29,37^. A high amount of WNV RNA – approx. 100-fold higher than in any of the positive plasma samples investigated during this study – was also detected in urine of WNND patient Pa2/15. In follow-up investigations, WNV RNA was detected in urine samples of this patient for a period of 90 days after onset of symptoms, supporting the finding that in some cases urine samples are a suitable material for WNV detection^29,37^. Several urine samples were available from horse Ho1/16, however, no WNV RNA was detected in them.

We were lucky to be able to investigate three WNVs originating from one farm, i.e., from horse Ho1/16 and mosquito pools Cx1/16 and Cx2/16. While two of them (Ho1/16 and Cx2/16) proved to be genetically closely related (as expected), mosquito pool Cx1/16 turned out to be different (Fig. 2). This finding confirms the hypothesis that a number of genetically slightly different WNV strains are circulating in the same area. Surprisingly, the amino acid sequence of horse Ho1/16 was 100% identical to the sequence of blood donor BD2/15 (Table 2) despite one year time difference. However, no relationship could be determined between these two cases except that they were probably infected in the same geographic area where the same virus strain was maintained in subsequent mosquito generations.

It is also worth mentioning that the cobas^®^ WNV test commonly used for screening blood donations for WNV nucleic acid is broadly reacting and detects also other flavivirus nucleic acids such as Usutu virus (USUV), as recently demonstrated in Austria in one blood donation from 2016 and in six donations from 2017^38^. In Austria and in several other European countries USUV and WNV are co-circulating in the same areas^39,40^.

In conclusion, WNV can be considered endemic in Vienna and its surroundings affecting humans, birds and horses. The virus circulates in *Culex* mosquitoes, in which it is also able to overwinter, as demonstrated in the Czech Republic close to the Austrian border^41^. Several genetic variants are co-circulating in eastern Austria, however all belong to WNV sublineage 2d. No association was found between known neuroinvasiveness/pathogenicity markers and clinical outcome of the infections, although all strains showed the genetic potential of causing neurological disease. Disease severity was more related to host factors such as higher age in humans and increased vulnerability of certain species of birds. However, the genetic markers along with other mutations allowed a phylogenetic subclustering of the analyzed WNV strains.

This paper demonstrates the advantages of integrated human-animal-vector surveillance for WNV. The integrated activities are based on the close collaboration between regional institutions and authorities involved in human, animal, and environmental health. Such an approach allows early detection of seasonal WNV circulation in an affected environment and subsequent prompt application of appropriate measures to protect both human and animal populations^42^. Vaccination is the primary method for reducing the risk of infection with WNV. While vaccines against WNV for horses are commercially available^43^, for humans they are still in development^44^. Therefore, other preventive public health measures, such as individual prevention to mosquito bites and possible reduction of breeding habitats, are advised to the public during peak WNV activity. In Austria, the information gathered is continuously published on the AGES homepage^45^. As shown by an Italian surveillance study, the virus activity in an environment was observed well in advance respect to the appearance of the first human case^46^. This observation could not be directly confirmed in our study, because most of the human and animal WNV infections occurred during peak mosquito activity between end of July and middle of September each year (Table 1). Nevertheless, WNV was detected in mosquitoes collected not only during their activity season but also in the late-autumn and winter months signaling virus activity in the corresponding areas.

Furthermore, after the first seasonal detection of WNV in mosquitoes or birds, blood donations originated from the affected areas are routinely screened. This definitely decreases transmission risk and consequently results in a significant reduction of health care costs^47^. Lastly, sufficient knowledge of currently circulating human and animal virus strains allows reasonable adaptation of applied procedures for more sensitive and specific virus detection in future.

Integrated surveillance measures provide a comprehensive description of arbovirus activity in a given region. Early detection of the introduction of newly circulating viruses or viral variants is the goal of arbovirus surveillance, which frequently only relies on vector surveillance. In addition to case reports and disease incidence rates, detailed genetic analyses of detected viruses may offer in-depth insight into virus evolution and emergence. These data facilitate proactive public health measures to prevent or mitigate arbovirus outbreaks in a region.
